# Revealing the ultrastructure of live *Candida albicans* using stimulated emission depletion microscopy

**DOI:** 10.1098/rsob.250031

**Published:** 2025-10-22

**Authors:** Katherine J. Baxter, Liam Mark Rooney, Shannan Foylan, Gwyn W. Gould, Gail McConnell

**Affiliations:** ^1^ Strathclyde Institute of Pharmacy and Biomedical Sciences, University of Strathclyde, Glasgow, UK

**Keywords:** Candida, live cell, ultrastructure, fluorescence, lipids, microscopy

## Abstract

*Candida albicans*, a commensal fungal pathogen, is a leading cause of opportunistic infections in immunocompromised individuals. High-resolution imaging of its cellular architecture is essential for uncovering pathogenic mechanisms and identifying antifungal targets. Here, we applied live-cell stimulated emission depletion (STED) microscopy to visualize subcellular structures in *C. albicans* with nanoscale precision. We found that fluorophores commonly used for STED imaging in mammalian cells were ineffective in *C. albicans*. In contrast, Nile Red enabled robust labelling of dynamic lipid-rich structures, including lipid droplets and intracellular membranes, with sufficient signal for long-term imaging. Using Nile Red with STED microscopy, we achieved over threefold improvement in lateral resolution (approx. 85 nm) compared to confocal microscopy, with minimal photobleaching even during continuous time-lapse acquisition over 8 h. Notably, dynamic tracking of lipid droplets revealed heterogeneous mobility patterns across individual cells, suggesting functional compartmentalization. Our findings establish STED microscopy with Nile Red staining as a powerful approach for visualizing dynamic ultrastructure in live fungal cells, expanding the toolkit for high-resolution fungal cell biology and offering new avenues for studying fungal pathogenesis and antifungal action at the nanoscale.

**Figure:**
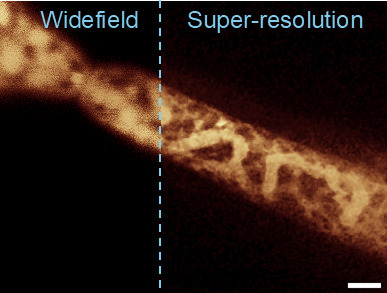

## 1. Introduction

*Candida albicans* is a pleomorphic fungus that plays a significant role in human health, particularly within microbial communities of the mouth, gut and other mucosal surfaces [1]. As a part of the normal microbiota, *C. albicans* can exist harmlessly, aiding in nutrient processing and competing with harmful pathogens, which helps maintain a balanced microflora. However, *Candida* species are also opportunistic pathogens [1]; when the immune system is weakened or the host experiences dysbiosis (e.g. due to illness), *C. albicans* can lead to acute or invasive candidiasis [2]. These infections range from mild, superficial issues like oral thrush [3] or fungal foot and nail infections [4] to severe, systemic infections that can be life-threatening, particularly in immunocompromised individuals [5].

One of the key critical structural features of *C. albicans* is its ability to switch between different forms, namely yeast, pseudohyphal and hyphal, which allows it to spread and invade tissues effectively [6]. The complex cell wall of *C. albicans*, composed of β-glucans, chitin and mannoproteins, provides protection and enables it to adhere to host cells, a necessary step for colonization [7]. *C. albicans* can also form biofilms, dense layers of cells encased in a protective matrix, especially on medical devices, making these infections more persistent and resistant to antifungal treatments [8]. Additionally, the genetic adaptability and ability to secrete enzymes that break down host tissues enhance the virulence of *Candida* species [9]. These structural features make *C. albicans* a resilient pathogen, challenging to treat and control, especially in immunocompromised patients. Understanding these structural components is essential in developing effective antifungal therapies and preventive strategies.

Electron microscopy (EM) has been essential for high-resolution imaging of *C. albicans* ultrastructure, but it poses significant limitations, especially for live-cell studies. Standard EM techniques require fixation, dehydration and complex sample preparation steps that kill cells and introduce artefacts, making real-time observation of dynamic processes, such as lipid droplet trafficking, impossible. Moreover, EM typically lacks molecular specificity and offers only static structural information from small sample areas. Its high cost, operational complexity, and the need for a vacuum environment further reduce its suitability for large-scale or live-cell studies, even when advanced variants like cryo-EM are employed.

Fluorescence microscopy enables live-cell imaging and molecular specificity through fluorescent tagging, and this has greatly advanced our understanding of *C. albicans* biology, including morphological transitions and intracellular signalling. However, conventional fluorescence microscopy is fundamentally limited by the diffraction of light, which restricts resolution to around 200−300 nm laterally and 500−700 nm axially, which is insufficient to resolve many fine subcellular structures. As a polymorphic fungal pathogen, *C. albicans* undergoes complex morphological transitions and exhibits highly dynamic behaviours that are central to its pathogenicity, including hyphal growth, organelle trafficking and cell wall remodelling. These processes typically occur at the nanoscale, below the diffraction limit of traditional fluorescence microscopy.

To overcome these limitations, super-resolution microscopy techniques have been developed to surpass the diffraction limit of light, offering much higher resolution—down to approximately 20 nm. These methods, including stimulated emission depletion (STED), structured illumination microscopy (SIM), photoactivated localization microscopy (PALM), super-resolution through radial fluctuations (SRRF) and stochastic optical reconstruction microscopy (STORM), each employ unique strategies to achieve nanoscale imaging. STORM microscopy has been used previously to explore the fine structure of beta-glucan exposed on the cell wall of *C. albicans* before and after antimycotic drug treatment, but this method is best applied to the study of sparse structures [10]. SRRF has been used to study GPI transamidase in the yeast-form of *C. albicans*, but the resolution was not reported, and the computational method introduced grid artefacts into image data. STED microscopy was selected for this study due to its fast acquisition speed and compatibility with live-cell imaging. By selectively depleting fluorescence around a focal point, STED achieves high spatial resolution, making it especially valuable for visualizing dynamic nanoscale processes in living cells. Despite its potential, STED has not yet been widely applied to study the ultrastructure of live *C. albicans*.

**Figure 1.**
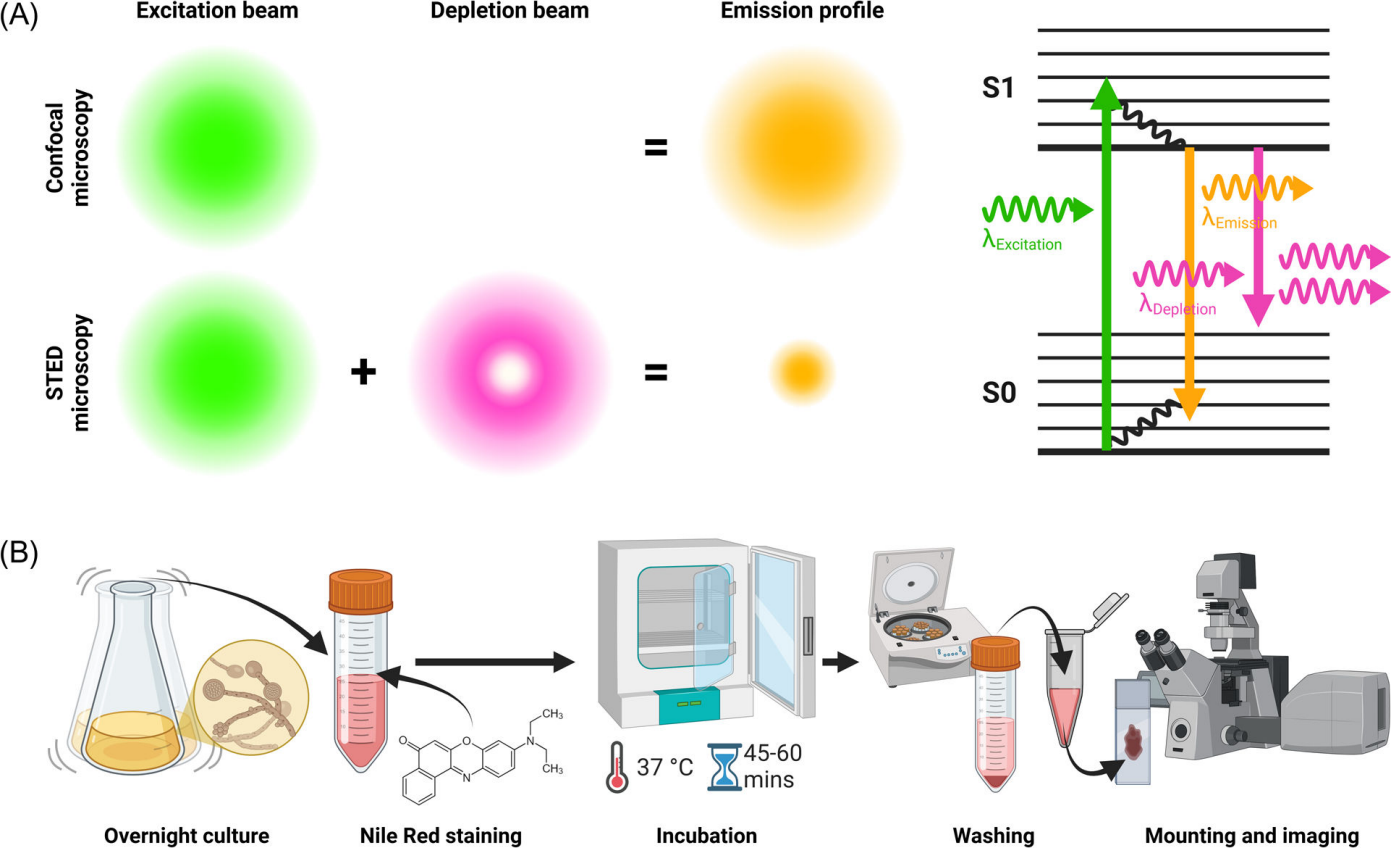
(A) A comparison of the emission profile acquired using confocal laser scanning microscopy (CLSM) and STED microscopy, along with a Perrin–Jablonski diagram describing the emission depletion during the fluorescence process. A depletion beam is aligned with the excitation laser during STED imaging to deplete the emission of fluorophores in the depletion region, resulting in a smaller overall emission profile and enhanced spatial resolution. Disruption of the internal conversion process causes relaxing electrons to immediately return from the excited state (S1) to the ground state (S0), releasing a red-shifted photon which is blocked by the microscope fluorescence emission filters, while fluorescence from S1 to S0 is collected from the confined excitation volume and used to form an image. (B) A methods workflow showing the growth, staining and preparation methods for live-cell STED imaging of *C. albicans*.

In this study, we have developed a robust protocol for live cell staining and time-lapse STED microscopy of *C. albicans*, enabling the visualization of dynamic cellular components, including organelles, specifically mitochondria and lipid droplet, distribution (figure 1). The STED principle, shown in figure 1A, allowed us to resolve features beyond the diffraction limit of conventional light microscopy, offering new insights into the ultrastructure of *C. albicans* without the strenuous specimen preparation requirements of electron microscopy. Our approach highlights the potential of super-resolution imaging in fungal research, providing a clearer picture of the cellular machinery of *C. albicans* and advancing our understanding of the role this important pathogen plays in human health and disease.

## 2. Methods

### 2.1. Specimen preparation

To initiate *C. albicans* culture, a single colony was inoculated into 5 ml of Luria–Bertani (LB) medium supplemented with 0.2% glucose and incubated overnight at 37°C for approximately 16−18 h. The duration of incubation influenced the extent of hyphal formation, with longer incubation times promoting more hyphal growth. After incubation, the culture was examined under a light microscope to assess the extent of hyphal development.

The effectiveness of multiple different stains for STED microscopy of live *C. albicans* were assessed, actin LIVE 460L (Abberior, Germany), DNA LIVE 590 (Abberior, Germany) and Nile Red (Sigma Aldrich, UK). These dyes were chosen because of their proven compatibility with STED microscopic imaging of mammalian cells [11–13] using 488 and 561 nm excitation, respectively, and depletion at 775 nm. Both actin LIVE 460L and DNA LIVE 590 are optimized for live cell imaging. Nile Red is a commonly used lipophilic fluorescent stain, used for both live and fixed cell imaging [14].

### 2.2. Staining

Abberior LIVE stains were prepared as described on the manufacturer’s website (https://abberior.rocks/), with lyophilized stains resuspended at a concentration of 1 mM in 100% DMSO (Sigma Aldrich, UK). Immediately prior to use, LIVE stain stocks were diluted to a final concentration of 0.1 µM in 200 µl of fresh, pre-warmed LB supplemented with 0.2% glucose. To stain *C. albicans,* 200 µl of overnight culture in an Eppendorf tube was spun at 2500 rpm for 2 min in a microfuge (Centrifuge 5418, Eppendorf) to pellet the cells, before resuspension in the 200 µl of pre-warmed LB containing 0.1 µM of stain. The Eppendorf tube containing cells and stain was then shielded from light and placed inside a universal tube for incubation at 37°C for 45–60 min at 220 rpm, prior to mounting.

For staining with Nile Red, 45 µl of PBS (pH 7.3) was added to a 55 µl aliquot of 90 µg ml^–1^ Nile Red in 100% dimethylsulfoxide (DMSO) and mixed thoroughly, ensuring that the solution was kept in the dark to protect the dye from degradation. A 200 µl aliquot of the overnight *C. albicans* culture was transferred to an Eppendorf tube, followed by the addition of 20 µl of the Nile Red solution in 55% /45% DMSO/phosphate-buffered saline. The mixture was gently vortexed to ensure thorough mixing and to facilitate the staining of the cells. The Eppendorf tube was incubated as described above.

After incubation, Eppendorf tubes were removed from the universal tube and centrifuged (Centrifuge 5418, Eppendorf) at 2500 rpm for 2 min to pellet the cells. The supernatant, containing the staining solution, was carefully removed, and the pellet was resuspended gently in 200 µl of PBS (pH 7.3) to wash the cells and remove excess dye. An experimental workflow is presented in figure 1B. A minimum of three biological replicates were imaged for each live stain.

### 2.3. Specimen mounting

For mounting the stained cells, a 4-µl volume of the resuspended cell sample was directly applied onto coverslips (22 × 22 mm, thickness no. 1.5, VWR). Cells were immobilized by the addition of 8 µl of molten 0.5% agar spotted onto the cell sample and immediately sandwiched with a glass slide to create a thin agar layer. Microscope slides were inverted onto the specimens for imaging.

### 2.4. Microscopy

Imaging was performed using an inverted microscope (IX71, Olympus) with a manual stage and without autofocus, equipped with confocal laser scanning microscopy (CLSM) and STED capability (STEDYCON, Abberior Instruments). Imaging was performed at room temperature, and a round-microscope enclosure prevented ambient light from reaching the specimen during imaging.

A 488 nm laser for excitation of specimens stained with LIVE 460L, and a 561 nm laser was used for excitation of fluorescence from both LIVE 590 and Nile Red stained specimens while a third laser at a wavelength 775 nm performed the depletion needed for STED microscopy. Typical relative laser powers were approximately 2% at 561 nm for CLSM, and 10% for both the 488 and 561 nm lasers and 50% of the depletion laser for STED. These values were chosen to maximize imaging contrast at a relatively fast imaging speed. As all stains emitted fluorescence in the red region of the spectrum, a spectral detector spanning 575–625 nm was employed to minimize the contribution of backscattered laser light and improve image contrast for imaging of both LIVE stains and Nile Red stained specimens.

Images were acquired using a high numerical aperture (NA) oil immersion objective (100×, NA 1.4), with a 60 µm diameter pinhole. The theoretical lateral resolution (*r*) of a CLSM [15] is calculated from *r* = 0.61*λ*/NA to be 268 nm for an emission wavelength (*λ*) of 616 nm and an objective lens with NA = 1.4.

Images were obtained with a pixel size of 30 nm to satisfy the Shannon–Nyquist sampling criterion [16], with an image size of 100 × 80 µm, and a 10-µs pixel dwell time, with each image taking approximately 90 s to acquire. For time-lapse imaging, a 20-min interval between images was chosen, and images were recorded over a 10-h period. Images were acquired using STED microscopy and CLSM alternatively. Images were saved in 16-bit format using the proprietary .obf file type of the microscope.

### 2.5. Image processing

Images in .obf format were processed using FIJI (v1.54f) [17]. In some time-lapse recordings, the specimens drifted in the lateral plane during imaging: for these data, a manual drift correction was performed using FIJI with the ‘*Correct 3D drift*’ in-built plugin [18]. Default settings were applied except the number of pixels of *x* and *y* correction were set to a maximum shift of 30 pixels each, and data were corrected in in the *x* and *y* directions only. The contrast of CLSM datasets was adjusted for the purpose of presentation using CLAHE [19], with default settings applied. No contrast adjustment was performed to STED microscopy datasets. Image data were converted to OME TIFF format for study, and time-lapse recordings were resized and saved as .AVI files for display of movies.

### 2.6. Image data analysis

To measure the resolution of our images, we adopted two different methods. The first was to take a line ROI across the same region of a CLSM micrograph and a STED image, and to measure the separation between two finely spaced objects. However, this method introduces human bias. As such, we also applied image decorrelation analysis (IDA), which is based on partial phase correlation and does not rely on user-defined parameters, and assesses the resolution from the whole image [20]. The FIJI plugin ‘*Image Decorrelation Analysis*’ was used with default settings, using input data from both CLSM and STED microscopy. IDA returns the spatial frequency limit, which corresponds to the image resolution.

Lipid droplet trafficking was tracked using TrackMate [21,22], an openly available plugin for FIJI. Lipid droplets were detected using a *Laplacian of Gaussian* filter with an estimated object diameter limit of 0.35 µm, a quality threshold of 1966 and sub-pixel localization enabled, which proved effective in selecting liposome foci. A *Linear Assignment Problem* framework [23] was used to track the foci throughout the time series. The maximum distance for frame-to-frame linking was set to 0.6 µm. The maximum gap closing distance was set to 0.6 µm, with a maximum frame gap of two frames. The resulting tracks were filtered to include tracks with a linearity of forward progression score below 0.9 for further analysis, removing perfectly linear tracks that likely arose due to errant combined tracks from closely neighbouring tracks. Tracks were presented with a colour coding corresponding to the measured track duration over the time series and the track features were exported as .CSV files and transcribed for plotting and statistical analyses using GraphPad Prism (v. 8.0.2; Mathworks). Tracking and analysis were performed using a 64-bit Windows Server 2016 standard operating system (v. 1607) with two Intel® Xeon® Silver 4114 CPU processors at 2.20 and 2.19 GHz and 1.0 TB installed RAM.

## 3. Results

### 3.1. Dyes suitable for stimulated emission depletion microscopy of mammalian cells are ineffective for labelling of *Candida albicans*

**Figure 2.**
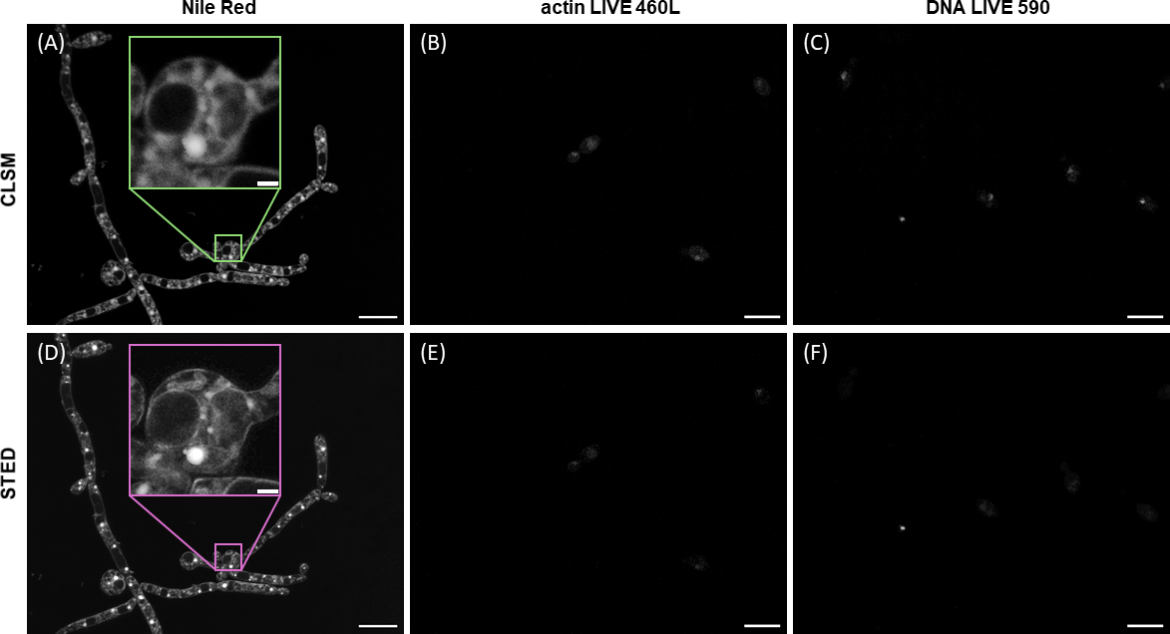
(A) *C. albicans* labelled with Nile Red, imaged with a CLSM. A digitally magnified ROI is shown with a green box, confirming intracellular staining. (B) *C. albicans* labelled with actin LIVE 460L, imaged with a CLSM. (C) *C. albicans* labelled with DNA LIVE 590, imaged with a CLSM. (D,E) and (F) show the STED counterparts of (A), (B) and (C). A digitally magnified ROI is shown in (D), showing the level of detail improvement possible with STED microscopy compared to CLSM. Scale bars = 10 µm.

Figure 2 shows images of *C. albicans* labelled with Nile Red obtained with (A) CLSM and (D) STED microscopy. Figure 2B*,*E shows CLSM and STED data obtained from *C. albicans* labelled with actin LIVE 460L, and figure 2C*,*F also shows CLSM and STED data, respectively, here with *C. albicans* labelled with DNA LIVE 590.

Fluorescence from the specimens labelled with Nile Red is clearly visible. Digitally magnified regions of interest (ROIs) are shown in the CLSM data in figure 2A and in the STED image data presented in figure 2D: in both cases, it is evident that Nile Red stained not only the cell boundary but also intracellular structures, including brightly stained structures representing lipid droplets. By comparison, virtually no fluorescence signal was visible in specimens labelled with either actin LIVE 460L or DNA LIVE 590.

### 3.2. Stimulated emission depletion microscopy reveals ultrastructure of live *Candida albicans* in specimens stained with Nile Red

**Figure 3.**
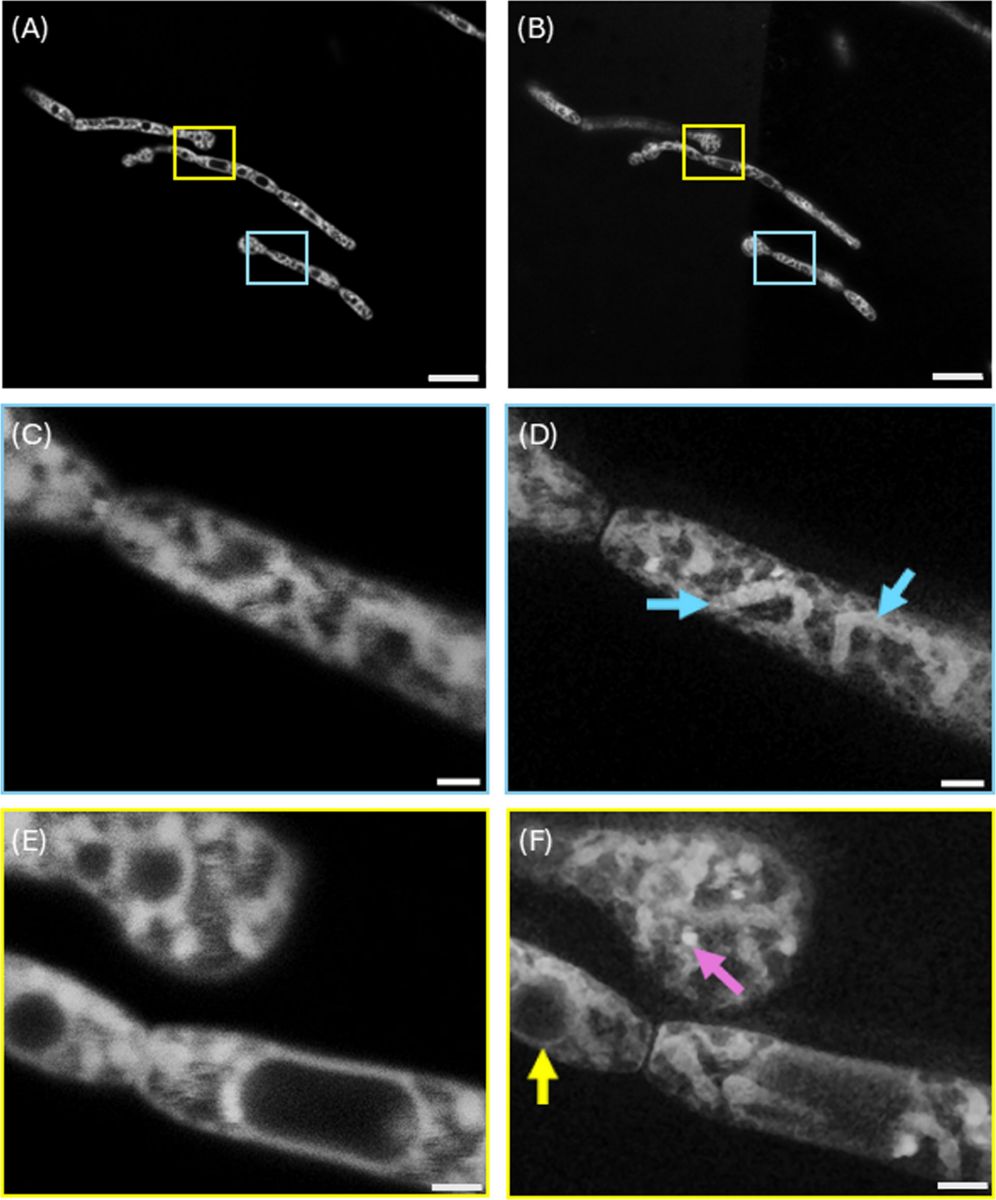
CLSM and STED microscopy of live *C. albicans* stained with Nile Red. (A) Representative confocal microscopy image. (B) Representative STED microscopy image of the same field of view as shown in (A). Cyan and yellow boxes highlight regions of interest (ROIs). Digitally magnified ROIs of CLSM data are shown in (C) and (E), while digitally magnified ROIs of STED data are shown in (D) and (F). Cyan arrows in (D) show mitochondria with the cristae visible, yellow arrow in (F) shows a vacuole interacting with mitochondria, and the magenta arrow in (F) highlights an example of a lipid-rich vesicle. The mitochondria and lipid droplets indicated by arrows in (D) and (F) are not clearly visible in the corresponding CLSM micrographs (C) and (E). Scale bars for (A ,B) = 10 µm, scale bars for (C–E) = 1 µm.

Figure 3 shows representative images of *C. albicans* stained with Nile Red imaged with CLSM and STED microscopy. Figure 3A shows a CLSM image, and figure 3B shows the equivalent STED microscope image. Two ROIs are highlighted with cyan and yellow boxes, and digital zooms of the CLSM and STED microscope images are shown in figure 3C and E and in figure 3D and F, respectively. There is a slight temporal offset of around 1 min between the CLSM and STED microscope images because they are acquired sequentially, which manifests as a slight disparity in the image content between the CLSM and STED micrographs, but with most of the structures retained.

A clear resolution improvement is shown in the STED microscopy images. Figure 3D shows the lipid-rich elongated structured characteristic of mitochondria [24] clearly in the STED data: the mitochondria are difficult to discriminate from other cellular structures in the corresponding CLSM data presented in figure 3C.

The STED data presented in figure 3F also reveal ultrastructure that is not visible in the confocal microscopy counterpart, figure 3E. Structures likely to be lipid droplets are visible: an example is highlighted with a magenta arrow in figure 3F. Membrane-bound vacuoles interacting with presumptive mitochondria are also observed, as indicated by the yellow arrow in figure 3F. These sub-diffraction limit-sized structures are not clearly visible in the CLSM data. While we cannot definitively identify mitochondria and lipid droplets in the absence of other organelle-specific stains, our proof-of-concept study shows that ultrastructure with properties akin to these organelles can be readily imaged. The design of new small STED-compatible organelle-specific markers will allow further progress.

### 3.3. Stimulated emission depletion microscopy of *Candida albicans* offers more than a threefold resolution improvement compared to confocal laser scanning microscopy

**Figure 4.**
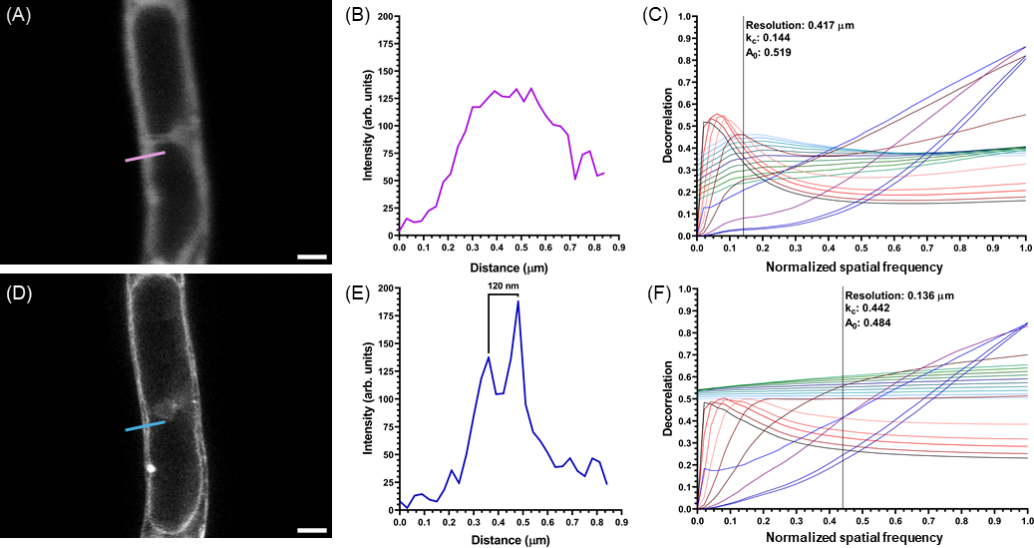
(A) CLSM image of *C. albicans* labelled with Nile Red. A green line shows a ROI. (B) Line intensity profile of the ROI shown in (A). (C) Image decorrelation analysis of (A) gives a resolution of 417 nm for this CLSM micrograph. (D) STED microscopy image of *C. albicans* labelled with Nile Red, showing the same ROI as (A) with a magenta line. (E) Line intensity profile of the ROI shown in (D) for the STED data. (F) Image decorrelation analysis of (D) gives a resolution of 136 nm for these STED data. Scale bars for (A) and (D) = 1 µm.

Resolution measurements of CLSM and STED data confirm the improvement in resolution using the STED imaging approach. CLSM and STED images of *C. albicans* labelled with Nile Red are shown in figure 4A,D, respectively, and a line ROI is shown in figure 4A. The same ROI was applied to figure 4D for the purpose of measuring the resolution of the image. As shown in figure 4B, it was not possible to resolve the structures highlighted in figure 4A. However, it was possible to resolve the discrete membranous structures in figure 4D, and the simple line ROI measurement produced a resolution measurement of 136 nm from the STED data, as shown in figure 4E,F. A direct comparison of the resolution of CLSM and STED microscopy is possible using IDA. The resolution of the CLSM and STED images shown in figure 4A,B was measured to be 417 and 136 nm, respectively, using IDA. While the resolution of the CLSM was measured to be poorer than predicted by the theoretical limit of diffraction, the STED microscope offered approximately a factor of two improvement in resolution compared to the theoretically best possible resolution of the CLSM [15], and more than a threefold improvement when comparing practical CLSM and STED microscopy, as shown in figure 4C,F.

A representative time-lapse recording of Nile Red stained *C. albicans* is presented as electronic supplementary material, video S1. The first frames of the movie for both the CLSM and STED data are shown in electronic supplementary material, figure S1, with digitally zoomed regions showing the level of improvement in detail using STED microscopy compared to CLSM. Both the CLSM and STED microscopy datasets showed organelles within approximately 8 h of the full 10.8-h long recording, indicating sustained cell viability. After approximately 8 h, a change in cell morphology was noted, with some arrest of motion across the field of view. This is likely a consequence of phototoxic effects, but these data nevertheless demonstrate the possibility for long-term imaging. However, only the high-resolution detail afforded by STED microscopy offered the possibility to measure and track sub-diffraction limited intracellular structures. Minimal photobleaching was observed in these long-term recordings. A small amount of specimen detachment from the coverslip was observed from *t* = 540 min in one part of imaged field, leading to a decrease in fluorescence signal and blurring of this region of the image, but the other cells in the specimen remained in focus throughout the full duration of the recording.

**Figure 5.**
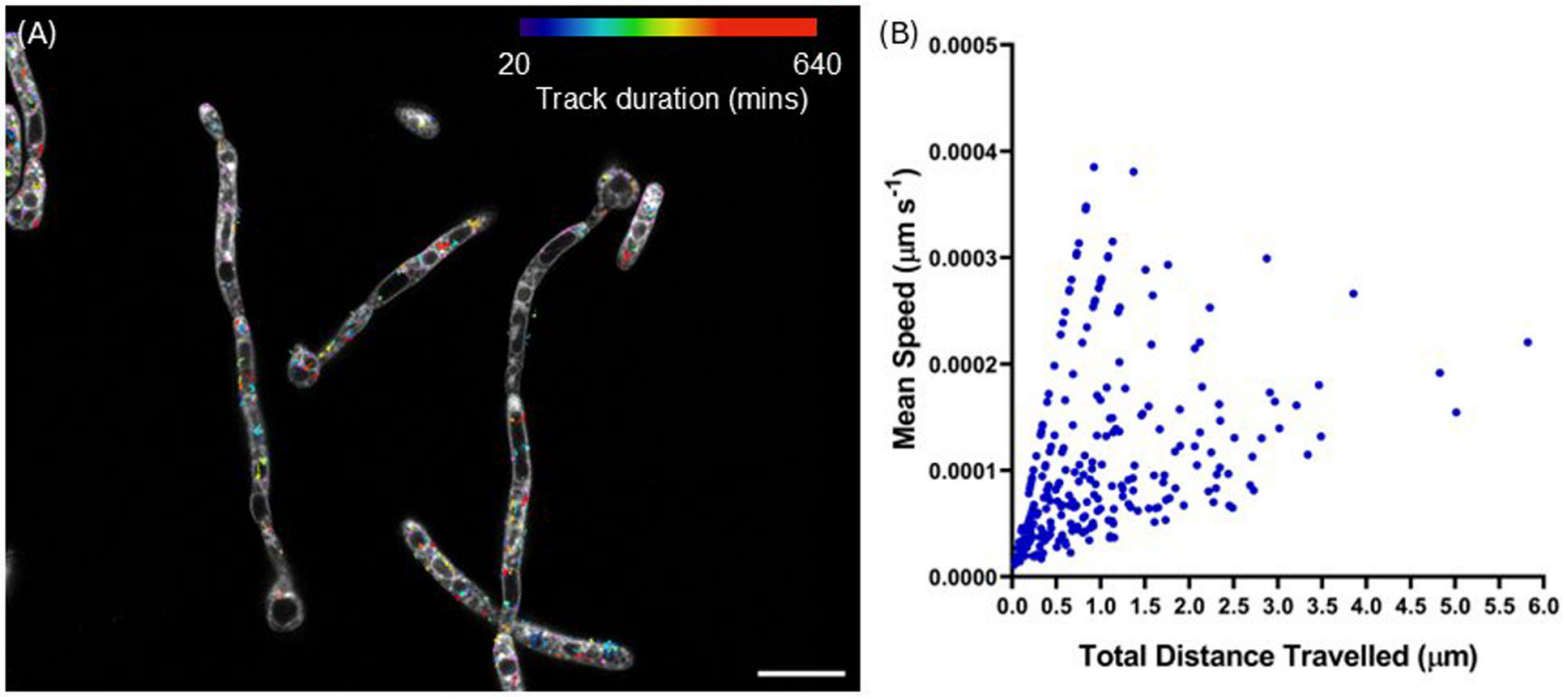
(A) An overlay of a STED image of *C. albicans* labelled with Nile Red and the tracks of lipid droplet trafficking over 12 h, with tracks colour-coded according to the measured track duration. (B) A plot showing the mean speed versus the measured distance travelled by each liposome during the time series shown in (A). Scale bar = 10 µm.

Figure 5 shows the tracking and quantification of lipid droplet trafficking in live *C. albicans* over 8 h acquired using STED microscopy. Tracks are presented in figure 5A, colour-coded by track duration. Lipid droplet trafficking was observed in multiple cells throughout the acquisition, with a mean track duration of 180 min (s.e. in the mean (s.e.m.) = 8 min 47 s, s.d. = 141 min 14 s). Figure 5B shows the relationship between lipid droplet trafficking speed and total distance travelled during the time series. In general, the mean speed of a given liposome trafficking event scaled with the length of the track, meaning longer tracks were typically slower than short tracks. The mean speed of lipid droplet trafficking was measured at 110.0 nm s^−1^ (s.e.m. = 5.2 nm s^−1^, SD = 83.0 nm s^−1^), with a maximum measured distance of 5.82 µm. Electronic supplementary material, video S2, shows the lipid droplet trafficking dynamics in a digitally magnified ROI from figure 5 and electronic supplementary material, video S1, overlayed with tracks colour coded as described for figure 5. The sub-cellular ultrastructure and organelle motility can be observed over an excerpt of 650 min from the full time series. In addition to enabling tracking of lipid droplet motility, the STED microscopy data better resolve the vacuolar membrane and provide clearer visualization of the vacuole itself, rather than inferring structure from the blur of the vacuole membrane and peripheral or interacting endosomes.

## 4. Discussion

In this study, we employed STED microscopy to investigate the ultrastructure of live *C. albicans*, and our findings showcase the feasibility of studying mitochondria and lipid droplets. The dynamic and regulated association between mitochondria and lipid droplets in mammalian cells is tightly linked to metabolic status [25], and thus our observation of close apposition of lipid droplets and mitochondria in *C. albicans* suggest that this is an area worthy of further exploration.

We observed that some fluorescent labels designed for STED microscopy, while effective for the study of mammalian cells, were not suitable for this super-resolution optical imaging method when applied to *C. albicans*. Both LIVE stains, like Nile Red, have no charge [26], so their ineffectiveness in *C. albicans* is unlikely to be caused by polarity. The molecular weight of LIVE 460L and LIVE 590 are not published, but based on the STAR derivative structure, which is approximately 880 Da [27], they are likely to be larger than that of Nile Red, which is a fluorescent molecule with one of the lowest molecular weights (318 Da) [28]. The cell wall of *C. albicans*, which is around an order of magnitude thicker than the plasma membrane of mammalian cells [7], is likely to be preventing the ingress of LIVE 460L and LIVE 590. Given the challenges of labelling native proteins in *C. albicans*, largely due to the need for codon optimization [29], the use of Nile Red enabled high-resolution imaging.

Our work with Nile Red has proven the benefit of STED microscopy for studying the living ultrastructure of *C. albicans*, but this staining method labels only lipids and hydrophobic regions, including lipid bilayers and, notably in the context of this study, intracellular lipid droplets. Fixation and permeabilization in combination with immunofluorescence could potentially be used to verify the structures stained with Nile Red, however, fixation methods commonly distort tissue and may introduce artefacts that may complicate structural analysis.

Nile Red has been shown to exhibit PAINT-like properties through transient interactions with hydrophobic environments, as described previously [30]. We recognize that limited dynamic binding and unbinding may occur over extended imaging periods, but these are likely to be minimal, given multiple rigorous washing steps in our protocol to reduce the presence of unbound dye. As such, the contribution of these transient events to the final image signal is expected to be minimal, and they are unlikely to influence the outcome of STED imaging experiments.

Commonly used STED dyes at similar excitation wavelengths to those used in our study such as ATTO 647N, DyLight 650 and Alexa Fluor 647 have comparatively high molecular weights at 660, 620 and 1100, respectively. To take advantage of the molecularly selective affinity that fluorescent labelling confers, e.g. for multi-colour STED microscopy of organelles labelled with protein-specific dyes, alternative fluorescent dyes with lower molecular weights compatible with STED imaging may be needed. Such new stains would assist in verifying the structures stained with Nile Red and could open the possibility of studying other dynamic cellular processes in *C. albicans*, though we note that the broad emission spectrum of Nile Red in the visible spectrum means that dual-colour live STED microscopy would potentially be best achieved with a molecule excited at a wavelength longer than 650 nm. These include the study of cell division during morphogenesis, enabling the observation of septin filaments at the site of cell division [31], understanding the remodelling of vacuoles in response to environmental stressors or changes in nutrient availability [32], the role of extracellular vesicles in the modulation of host–pathogen interactions [33], and unveiling how adhesin expression changes in *C. albicans* during the formation of biofilms on the surface of medical devices [34]. There is also considerable potential to apply this methodology to the study of drug and toxin effector molecules, with improved prospects for characterizing how antifungal agents disrupt cellular processes [35].

While the 120-nm resolution of this imaging method offers a considerable improvement over conventional technologies such as CLSM, a much higher spatial resolution is theoretically possible with STED microscopy. The theoretical resolution of STED microscopy is primarily determined by the wavelength of the excitation light, the size of the fluorescence emitting region, and the intensity and size of the depletion beam, and can provide resolutions of between 20 and 50 nm [36]. However, to reach these very high resolutions, specific experimental conditions must be met. These include using a very high depletion laser power, which can be damaging to the specimen and can prohibit live cell imaging. For single-shot imaging, it may be possible to improve our reported resolution considerably using higher optical powers. It is possible that the relatively thick cell wall in *C. albicans* [7] is also scattering the ordinarily well-defined laser spots, causing defocus and a decrease in measured resolution. A possible solution—which has already been proven in the use of STED microscopy to the study of mammalian cell specimens—is the use of adaptive optics (AO). AO systems typically use deformable optical elements to dynamically adjust the optical path and correct distortions [37]. This correction helps maintain the high resolution achieved by STED microscopy [38], even in thicker or more scattering samples (e.g. tissues or complex biological environments), where traditional methods may struggle. This, however, increases both the cost and complexity of the instrumentation, and AO elements may require on-going calibration and optimization, which may not be compatible with live cell studies. SIM [39] may offer an alternative method to STED for super-resolution imaging of *C. albicans*, but in this method a minimum of nine images are required to improve the resolution by up to a factor of two. It is, however, possible that the thick cell wall of *C. albicans* may introduce considerable artefacts that could prevent the use of SIM.

STED microscopy enabled tracking of intracellular organelles with high precision, low specimen drift and minimal photobleaching over more than 8 h. Our data resolved organelle movement, in this case lipid droplets, occurring over distances of less than 2 µm or within tight confinement neighbourhoods. Such short trajectories would be almost impossible to measure with an CLSM because of the relatively poor resolution afforded by diffraction limited optics. The measured track distances broadly agree with the scale of *C. albicans* cells and previously reported velocimetry studies in mammalian cell biology [40,41]. Lipid droplet, vesicle and vacuole trafficking have been reported to play multiple roles in the virulence, pathogenesis, drug resistance and ageing of *Candida* species. Vacuolar dynamics are highly regulated during normal development and physiology, maintaining ion homoeostasis and promoting hyphal formation [42], with vacuole-mediated autophagy being linked with pathogenesis [43]. Moreover, lipid droplet formation has been well documented in yeasts, showing their accumulation over the lifespan of the organism [44,45] and vertical transfer through lineages of daughter cells [46]. However, the ability to track lipid droplets and vacuole dynamics over long periods has been hampered due to the inability to resolve such small structures in conventional diffraction-limited imaging. By providing means to resolve these organelles in both space and time, researchers may adapt our methods to study the role of lipids in pathogenesis and drug resistance; for example, in understanding dynamic morphological changes in the organization of the *Candida* vacuole that promote yeast-to-hyphae transitions [43], or in maintaining the specific protein and lipid composition of the membrane, facilitating tissue invasion and infection. Lipid droplet dynamics are also inherently linked to drug resistance and antimicrobial resistance [47], for example by trapping and occluding toxins in lipid droplets [48], thus minimizing the efficacy of antifungal treatments.

We recognize, however, that the measured short track lengths may be the result of organelle motility, where organelles are moving out of the focal plane, or specimen drift. The limitations of two-dimensional tracking, whereby tracks may be mis-assigned if objects move in or out of the focal volume, may be overcome by use of three-dimensional STED acquisitions. However, three-dimensional STED exhibits intrinsically higher photobleaching due to increased power requirements, and there is often a compromise in lateral spatial resolution to accommodate enhanced *z*-resolution. This could be explored further through live-cell fluorescence recovery after photobleaching measurements.

Our results show the benefits of two-dimensional STED for super-resolved imaging of *C. albicans*, but three-dimensional STED is also possible [49]. Three-dimensional STED microscopy offers significant benefits over two-dimensional STED microscopy, particularly for studying complex three-dimensional structures within biological samples. Three-dimensional STED would offer insights into hyphal formation by providing high-resolution imaging of the hyphal tip [50], visualization of protein trafficking [51] and the distribution of signalling molecules during cell adhesion [52]. However, three-dimensional STED has considerable limitations, including high rates of photobleaching and phototoxicity that can compromise live cell studies. Moreover, the additional time required to scan in the axial direction for volumetric imaging may lead to difficulties in object tracking.

While we have demonstrated our method in *C. albicans*, our work raises the possibility to apply STED microscopy to other *Candida* species, such as *C. glabrata* or *C. auris. C. glabrata* exhibits resistance to common anti-fungal treatments (e.g. fluconazole) [53], and *C. auris* is a healthcare-acquired multi-drug-resistant pathogen recalcitrant to various anti-fungal treatments and decontamination methods [54] . Our method could be applied to investigate how drug resistance is mediated at the molecular level in both species, for instance, by examining changes in vacuole architecture or subcellular distribution of drug efflux pumps [55]. Moreover, our method could be adapted to the study of polymicrobial specimens and interkingdom interactions, such as those between *C. albicans* and bacteria including *Staphylococcus aureus*, e.g. to identify localized regions of signalling molecule accumulation, e.g. farnesol, within co-cultures [56]. This would provide valuable insight into how *C. albicans* communicates with other microbes or how it modulates its own behaviour and virulence in response to signals from other species.

## Data Availability

Datasets currently available for download at University of Strathclyde database [57]. Supplementary material is available online [58].
